# Adipokines, Hepatokines and Myokines: Focus on Their Role and Molecular Mechanisms in Adipose Tissue Inflammation

**DOI:** 10.3389/fendo.2022.873699

**Published:** 2022-07-14

**Authors:** Yakun Ren, Hao Zhao, Chunyan Yin, Xi Lan, Litao Wu, Xiaojuan Du, Helen R. Griffiths, Dan Gao

**Affiliations:** ^1^ Institute of Molecular and Translational Medicine, Xian Jiaotong University Health Science Center, Xi’an, China; ^2^ School of Basic Medical Sciences, Xi’an Jiaotong University Health Science Center, Xi’an, China; ^3^ Department of Pediatrics, The Second Affiliated Hospital of Xi’an Jiaotong University, Xi’an, China; ^4^ Department of Biochemistry and Molecular Biology, School of Basic Medical Sciences, Xi’an Jiaotong University Health Science Center, Xi’an, China; ^5^ Swansea University Medical School, Swansea University, Swansea, United Kingdom; ^6^ Department of Human Anatomy, Histology and Embryology, School of Basic Medical Sciences, Xi’an Jiaotong University Health Center, Xi’an, China

**Keywords:** adipokines, hepatokines, myokines, adipose tissue, inflammation

## Abstract

Chronic low-grade inflammation in adipose tissue (AT) is a hallmark of obesity and contributes to various metabolic disorders, such as type 2 diabetes and cardiovascular diseases. Inflammation in ATs is characterized by macrophage infiltration and the activation of inflammatory pathways mediated by NF-κB, JNK, and NLRP3 inflammasomes. Adipokines, hepatokines and myokines — proteins secreted from AT, the liver and skeletal muscle play regulatory roles in AT inflammation *via* endocrine, paracrine, and autocrine pathways. For example, obesity is associated with elevated levels of pro-inflammatory adipokines (e.g., leptin, resistin, chemerin, progranulin, RBP4, WISP1, FABP4, PAI-1, Follistatin-like1, MCP-1, SPARC, SPARCL1, and SAA) and reduced levels of anti-inflammatory adipokines such as adiponectin, omentin, ZAG, SFRP5, CTRP3, vaspin, and IL-10. Moreover, some hepatokines (Fetuin A, DPP4, FGF21, GDF15, and MANF) and myokines (irisin, IL-6, and DEL-1) also play pro- or anti-inflammatory roles in AT inflammation. This review aims to provide an updated understanding of these organokines and their role in AT inflammation and related metabolic abnormalities. It serves to highlight the molecular mechanisms underlying the effects of these organokines and their clinical significance. Insights into the roles and mechanisms of these organokines could provide novel and potential therapeutic targets for obesity-induced inflammation.

## 1 Introduction

Obesity has become a prominent health problem globally and is closely associated with many chronic diseases such as type 2 diabetes mellitus (T2DM), cardiovascular diseases, and certain types of cancer (1). Obesity develops when energy intake exceeds energy expenditure and is characterized by the excessive accumulation of adipose tissue (AT). When AT reaches its maximum capacity for energy storage, it releases free fatty acids, causing ectopic lipid deposition in other tissues such as the liver, skeletal muscle, and vasculature. This contributes to the development of metabolic diseases. Nonetheless, the molecular basis for the link between obesity and metabolic diseases remains unclear.

Gowning evidence suggests that chronic inflammation plays a critical role in mediating the pathogenic link of obesity with insulin resistance and T2DM. During the development of obesity, AT shows increased macrophage infiltration (2–4), with greater pro-inflammatory M1 activation as opposed to the M2 phenotype in lean subjects (5, 6). Consequently, these AT macrophages secrete high levels of pro-inflammatory cytokines, resulting in obesity-associated chronic low-grade inflammation (meta-inflammation) and impairments in insulin signaling (7). The triggers for macrophage accumulation in AT during obesity are still unclear, although adipocyte death and hypoxia have been proposed as potential contributors (8). Intracellularly, several pathways have been hypothesized to induce the pro-inflammatory activation of macrophages in AT including the Toll-like receptor (TLR4)/nuclear factor-k-gene binding (NF-κB) pathway, c-Jun N-terminal kinase (JNK) pathway and caspase/NLRP3 inflammasome (9). These inflammatory pathways could act as new treatment targets for obesity-associated metabolic disease.

In addition to regulating energy homeostasis, AT also functions as an endocrine organ and releases various bioactive molecules (called adipokines). These molecules are involved in the regulation of various biological processes, such as inflammation, insulin resistance, and glucose and lipid metabolism (10). Similar to AT, the liver and skeletal muscles also function as endocrine organs, producing proteins called hepatokines and myokines, respectively (11). These organokines can mediate the crosstalk between various tissues *via* endocrine, paracrine, and autocrine pathways. So far, AT has been found to secrete more than 600 proteins, and only a small proportion of these has been investigated (12, 13). Obesity results in significant changes in the adipokine profile, creating a shift towards elevated pro-inflammatory adipokines such as leptin and resistin and reduced levels of anti-inflammatory adipokines such as adiponectin and ZAG. Compared with adipokines, hepatokines and myokines have recently gained greater attention as regulators of metabolic diseases (14).

This review aims to provide an updated information on the role of adipokines, hepatokines, and myokines in the regulation of AT inflammation and related metabolic abnormalities. Further, it highlights the molecular mechanisms underlying of the effects of these organokines. An in-depth understanding of the role and molecular mechanism of these organokines could guide the identification of novel therapeutic targets for obesity-induced inflammation and metabolic diseases.

## 2 Adipokines

AT secretes a variety of bioactive substances that are associated with chronic inflammation, insulin resistance, and an increased risk of T2DM. Here, we review the current literature on adipokines with pro-inflammatory and anti-inflammatory properties and highlight their molecular roles in the obesity-related regulation of AT inflammation.

### 2.1 Pro-Inflammatory Adipokines

#### (1) Leptin

Leptin is a 16-kDa monomeric non-glycosylated protein that is primarily secreted by adipocytes. Circulating levels of leptin are directly proportional to body fat mass and reflect the body’s metabolic status. Leptin is upregulated upon food intake and in patients with obesity and decreases during fasting or weight loss. Leptin, the first classical adipokine, was discovered almost 30 years ago. It was initially considered a ‘satiety signal’ that regulates body weight by inhibiting food intake and stimulating energy expenditure *via* a feedback loop between adipocytes and hypothalamus (15).

Six leptin receptor isoforms (LepRs) have been identified, with the long isoform LepRb being highly expressed in the hypothalamus (16) and transducing the intracellular leptin signaling. After binding to LepRb, leptin activates the JAK2-STAT3 pathway and induces the expression of the suppressor of cytokine signaling 3 (SOCS3) — a negative regulator of leptin signaling — and the and hypothalamic anorexigenic neuropeptide proopiomelanocortin (POMC). Meanwhile, it downregulates the orexigenic neuropeptide Y (NYP) and agouti protein (AgRP) (17). Mice with leptin deficiency (*ob/ob*) or leptin signaling deficiency (*db/db*) develop severe obesity due to hyperphagia (18). However, only a small proportion of obesity cases in humans are associated with leptin deficiency. However, majority patients with obesity show higher leptin levels due to the development of hypothalamic leptin resistance (19). Similarly, diet-induced obese (DIO) mice also develop leptin resistance centrally (20). Hence, improvements in central leptin sensitivity could be effective in tackling for general obesity. The role of leptin in the central nervous system has been reviewed extensively before (21).

In addition to its role in energy balance, leptin also acts as an immunomodulatory cytokine, regulating immune function and inflammatory responses (22). Leptin receptors are also expressed in immune cells, and macrophages express the long form LepRb (23). Early studies demonstrated that both *ob/ob* and *db/db* mice show impaired immunity and increased susceptibility to infections (24), suggesting that leptin is required for maintaining normal immune function. However, in obese individuals, high leptin levels seem to play a pro-inflammatory. For example, in one study, *in vitro* leptin treatment promoted pro-inflammatory cytokine TNF-α secretion in macrophages *via* p38 and the JNK MAPK pathway (25, 26) and enhanced CCL chemokines expression *via* the JAK2/STAT3 pathway (27). Moreover, leptin acts as a potent chemoattractant for monocytes and macrophages *via* the LepRb-mediated activation of the JAK/STAT, MAPK and PI3K pathways (23). Furthermore, the intraperitoneal injection of leptin significantly induces neutrophil migration to the peritoneal cavity and enhances the biogenesis of lipid droplets in peritoneal macrophages in both lean and high-fat diet (HFD)-induced obese mice (20). It is believed that leptin promotes lipid accumulation in macrophages *via* the activation of the PI3K-AKT-mTOR pathway (28). Lipid accumulation reprograms AT macrophages to a unique metabolic activation phenotype that produces high levels of inflammatory cytokines (29). In addition, chronic leptin treatment promotes preadipocyte differentiation and the secretion of pro-inflammatory cytokine TNF-α from adipocytes (30). These studies suggest that leptin plays an immunometabolic role in local AT during obesity (31). Further studies are required to understand its precise role and signaling effects in human AT and human obesity.

#### (2) Resistin

Resistin is a 12.5-kDa cysteine-rich peptide, which was initially identified as a protein secreted by murine white adipocytes after treatment with antidiabetic thiazolidinedione drugs. Resistin was named so because it induced insulin resistance in mice and was hypothesized to be the molecular link between obesity and diabetes (32). In humans, resistin is predominantly expressed in peripheral blood mononuclear cells (PBMC) and macrophages (33). There are considerable differences in resistin expression and regulation between mice and humans. Mouse adipocytes show a genomic binding site for the nuclear receptor PPARγ, which controls the expression of the resistin gene. In contrast, human adipocytes lack this binding site (34). Consequently, resistin expression is prominent in white adipocytes in mice but is predominantly found in resident inflammatory cells in human AT (35). In 3T3-L1 adipocytes, resistin gene expression is suppressed by insulin and TNF-α, while it is induced by high glucose and dexamethasone treatment (36). By contrast, in human immune cells, resistin expression is markedly induced by various inflammatory stimuli, including LPS, TNF-α, IL-6, and IL-1β (37).

Circulating resistin levels are elevated in mouse models of diet-induced and genetic obesity (32), suggesting that elevated resistin levels may be closely related to obesity and associated metabolic dysfunctions. *In vitro* recombinant resistin treatment induces insulin resistance in a variety of cells, including adipocytes, skeletal muscle cells, and hepatocytes (32). Conversely, the neutralization of resistin with anti-resistin antibodies improves insulin sensitivity in DIO obese mice and 3T3-L1 adipocytes (32). Further, the knockdown or deletion of resistin increases insulin sensitivity in HFD-fed obese mice (38). However, in humans, the association between serum resistin levels and metabolic diseases remains under debate (39). This could be due to the differences in resistin sources between humans and mice, and therefore, more epidemiological studies are required.

In addition to peripheral metabolic tissues, resistin also targets immune cells, thus playing a significant regulatory role in human inflammatory responses. For example, *in vitro* treatment with human resistin upregulates pro-inflammatory cytokines such as TNF-α and IL-12 in human macrophages *via* the activation of the nuclear factor-κB (NF-κB) signaling pathway (40). Moreover, humanized-resistin transgenic mice that lack adipocyte-derived mouse resistin but have macrophages that produce human resistin develop white AT inflammation and insulin resistance after 3 weeks of HFD consumption (41). This suggests that human resistin acts similarly to murine resistin and could play an important role in the early onset and progression of obesity and insulin resistance. Several studies have highlighted the pro-inflammatory effect of the resitin-TLR4 axis. Mechanistically, resistin binds to TLR4 and activates NF-κB in human macrophages and mouse hypothalamic cells *via* the JNK and p38 MAPK pathways (42, 43). Given its role in insulin resistance and inflammation, resistin could mediate inflammation in obesity and explain the relationship between obesity and associated metabolic diseases. However, the identification of a specific receptor for human resistin could provide a better understanding of its molecular roles in human obesity and metabolic diseases.

#### (3) Chemerin

Chemerin is a 14-kDa secreted chemotactic protein that is highly expressed in white AT. Chemerin levels are elevated both systemically and in adipocytes in patients with obese and metabolic syndrome (44, 45). By binding to its G protein-coupled receptor, chemokine like receptor-1 (CMKLR1), chemerin regulates adipocyte differentiation. More importantly, it recruits circulating plasmacytoid dendritic cells (pDCs) to visceral AT (46), initiating an innate immune response and contributing to AT inflammation in obesity. Mechanistically, pDCs are activated by the AT-derived high-mobility group B1 (HMGB1) protein *via* RAGE and TLR9 and produce the type I interferons (IFNs), which in turn promote the pro-inflammatory polarization of AT macrophages (46). Accordingly, CMKLR1 deficiency protects mice from HFD-induced obesity, AT inflammation, and insulin resistance (47). In addition, chemerin can also bind to an atypical chemokine receptor, chemokine receptor-like 2 (CCRL2), whose deficiency accelerates HFD-induced obesity and insulin resistance by promoting macrophages infiltration in AT (48). Hence, CCRL2 may serve as a competitive receptor for chemerin. Recently, it was found that the chemerin-CMKLR1 axis negatively regulates cold-induced thermogenesis in beige fat by interrupting type 2 innate immune responses in AT (49). Collectively, elevated chemerin levels play a critical pathological role in obesity. Hence, the adipose chemerin-CMKLR1 pathway could be a potential therapeutic target for obesity-related metabolic disorders.

#### (4) Progranulin

Progranulin (PGRN) is an ~88-kDa glycoprotein known as granulin/epithelin precursor (GEP) or PC cell-derived growth factor. It is widely expressed in a variety of cell types, including epithelial cells, fibroblasts, myelogenous cells, and adipocytes. PGRN can directly bind to TNFRs and prevent the interaction between TNF-α and TNFR. Therefore, it has been proposed to function as an anti-inflammatory protein in arthritis (50). Moreover, PGRN is a key adipokine that mediates HFD-induced obesity and insulin resistance *via* IL-6 in AT (51).

Circulating progranulin levels are higher in patients with obesity comorbid with insulin resistance or T2DM (52, 53) and are positively associated with macrophage infiltration in the omental AT of subjects with T2DM (54). Moreover, PGRN expression is also upregulated in the omental AT of subjects with metabolic syndrome and mediates insulin sensitivity (53). Progranulin expression is induced during adipocyte differentiation and is promoted by classical inflammatory stimuli such as LPS, TNF-α and IL-6 as well as ligands for TLR1/2 and TLR2/6 in mouse 3T3-L1 adipocytes (55). This suggests that PGRN could play a protective role against innate immune responses, in situations such as infection by gram-positive bacteria.

Li et al. studied the effects and molecular mechanisms of PGRN in AT inflammation and insulin resistance (56). PGRN knockdown partially improved insulin signaling and decreased JNK activation and ERK phosphorylation in 3T3-L1 adipocytes exposed to TNF-α. Meanwhile, PGRN treatment resulted in glucose intolerance and insulin resistance as well as an increase in AT macrophage infiltration and inflammation. However, these effects of PGRN were attenuated by the blockade of NF-κB and overexpression of sirtuin 1 (SIRT1). These results suggested that PGRN plays a pro-inflammatory role in adipose inflammation and insulin resistance, and this effect is partly mediated *via* SIRT1-NF-κB signaling. Further studies are required to identify the receptors of PGRN and its downstream signaling pathways. Nevertheless, PGRN may be a novel therapeutic target for chronic inflammation and insulin resistance in obesity and T2DM.

#### (5) RBP4

Retinol-binding protein-4 (RBP4) is a 21-kDa protein that specifically transports retinol in the circulation and is mainly secreted by the liver and adipose tissue. A previous study showed that adipocytes are a major cell type expressing RBP4 in human subcutaneous AT compared to stromal vascular factions 57). The expression of RBP4 and its receptor STRA6 is upregulated by PPARγ agonists and metabolic stresses such as high- glucose and palmitate treatment (58, 59). In contrast, their expression in human adipocytes is downregulated by the pro-inflammatory cytokines TNF-α and IL-1β (60).

Using adipocyte-specific glucose transporter 4 (Glut4) knockout (KO) mice, a study showed that RBP4 functions as an adipokine negatively regulating insulin sensitivity (61). RBP4 expression is selectively upregulated in the AT of adipose-Glut4 KO mice, and serum RBP4 levels are also elevated in several insulin-resistant mouse models and human subjects. Moreover, the overexpression of RBP4 or recombinant RBP4 injection induces insulin resistance, whereas RBP4 deficiency increases insulin sensitivity. Mechanistically, RBP4 increases the expression of the major gluconeogenic enzyme phosphoenol pyruvate carboxykinase (PEPCK) in the liver and impairs insulin signaling in skeletal muscle (61). Although hepatocytes are thought to be a major site of RBP4 secretion (62), the hepatocyte-specific overexpression of RBP4 does not impair glucose homeostasis (63). This suggests that a modest increase in circulating RBP4 secreted by the liver dose not impair glucose homeostasis and adipocytes are greater contributors to higher circulating levels of RBP4 in obesity (64).

In addition to insulin resistance, elevated serum or adipose levels of RBP4 are also observed in subjects with morbid obesity and are positively correlated with BMI and markers of macrophages and inflammation (57, 65, 66). Several studies have suggested a direct role of RBP4 in promoting AT inflammation and lipolysis, which contributes to obesity-induced insulin resistance and liver steatosis. Lee et al. reported that mice showing adipocyte-specific overexpression of human RBP4 have greater AT inflammation, which is characterized by increased TNF-α levels and macrophage crown-like structures in AT. Such inflammation stimulates basal lipolysis in adipocytes and induces hepatic steatosis (64). Notably, there is no evidence of increased hepatic *de novo* lipogenesis or decreased hepatic free fatty acid oxidation and very-low-density lipoprotein secretion, suggesting that RBP4-induced liver steatosis mainly arises from the primary effects occurring in AT. Moreover, the *in vitro* RBP4 treatment of human adipocytes directly stimulates basal lipolysis and activates macrophages, promoting the release of the pro-inflammatory cytokine TNF-α, and primes the NLRP3 inflammasome in macrophages *via* TLR4 (66, 67). Together, these studies suggest that targeting RBP4 could help in reducing AT inflammation and insulin resistance in patients with obesity and diabetes.

#### (6) WISP1

Wingless‐type (Wnt)-inducible signaling pathway protein-1 (WISP1, also known as CCN4) belongs to the Cyr61/CTGF/NOV (CCN) family of matricellular proteins and plays a role in cell adhesion, migration, differentiation, proliferation, and development (68). Widely expressed by a variety of tissues—including osteoblasts, cardiomyocytes, hepatocytes, neuronal cells, colon, lungs, and myocytes — WISP1 was recently validated as a novel adipokine released from human adipocytes and linked to inflammation and insulin resistance in obesity (69, 70).

Circulating WISP1 levels are elevated in obese subjects and are positively correlated with visceral adiposity, BMI, and systemic inflammation (71–73). WISP1 mRNA expression in both subcutaneous and visceral AT is positively associated with macrophage infiltration in AT and insulin resistance in healthy subjects. In contrast, weight loss reduces both circulating and AT levels of WISP1 (69). Moreover, circulating WISP1 levels are correlated with its expression in AT, suggesting that AT could be a major source of WISP1 production (69). Additionally, hepatic WISP1 expression is not upregulated in patients with NAFLD and shows no association with obesity parameters (69). Consistent with human studies, WISP1 mRNA and protein levels were upregulated in the epididymal AT of HFD-fed mice (69, 74), and this effect was potentially induced by elevated free fatty acids (74).

Adipocyte-secreted WISP1 has been suggested to play a pro‐inflammatory role in AT inflammation by directly activating macrophages. *In vitro* treatment with WISP1 stimulated the release of pro-inflammatory cytokines (e.g., TNF-α and IL-6) from human macrophages and promoted M1 macrophage polarization (69). In adipocytes, WISP1 neither induces cytokine expression nor affects preadipocyte differentiation and insulin signaling (69). In addition, a recent study reported that WISP1 contributes to obesity-induced liver steatosis and skeletal muscle insulin resistance *via* TLR4-mediated NF-κB- and JNK-dependent pathways 73, 74). This evidence supports the pathological role of WISP1 in obesity and its associated metabolic disorders. Transgenic mice with WISP1 mutations are required to verify its role in obesity and AT inflammation *in vivo*.

#### (7) FABP4

Fatty acid binding protein 4 (FABP4; also known as adipocyte protein 2, aP2) is a 14.6-kDa protein belonging to the FABP superfamily. It is highly expressed in AT and serves as an intracellular lipid chaperone for FFA import, storage and export (75). In addition to being one of the most abundant cytoplasmic proteins in adipocytes, FABP4 is also secreted from adipocytes (76), indicating its potential role as an adipokine. FABP4 expression is highly induced during adipogenesis and is transcriptionally regulated by PPARγ, (77). Moreover, its expression is also enhanced in macrophages derived from monocytes (78) and is induced by pro-inflammatory stimuli such as oxidized low-density lipoproteins and TLR agonists (79). FABP4 secretion from adipocytes is regulated by adenyl cyclase- protein kinase A (PKA)- and guanylyl cyclase-PKG-mediated lipolytic pathways (80), and to a less extent by microvesicles (81) and the lysosomal pathway (82). While elevated circulating FABP4 levels are a potent biomarker for obesity, insulin resistance, T2DM, and cardiovascular disease (83, 84), the levels of FABP4 in AT either show a negative association with adiposity and insulin resistance or no association at all (85, 86). This suggests that other tissues could be the primary contributors to elevated serum FABP4 levels in a state of obesity and insulin resistance. Indeed, a previous study reported that liver FABP4 mRNA levels are elevated in patients with obesity and insulin resistance as well as in *ob/ob* mice (86), suggesting that the liver could also be a source for circulating FABP4 levels in case of obesity.

Intracellularly, FABP4 has been shown to play a negative metabolic role in obesity and associated metabolic disorders *via* the regulation of lipolysis and lipogenesis (87). FABP4- deficient mice are protected from obesity-induced insulin resistance and hyperglycemia because of the increase in AT (88, 89), which results from reduced lipolysis and increased *de novo* lipogenesis, especially that of palmitoleate (90, 91), Moreover, FABP4 deficiency in macrophages also increases *de novo* lipogenesis, with increased palmitoleate production, which reduces lipid-induced endoplasmic reticulum (ER) stress (92). Mechanistically, FABP4 has been demonstrated to directly interact with HSL and thereby facilitate lipolysis *via* p38 (93, 94) and suppress PPARγ activity by inducing the proteasomal degradation of PPARγ (77). These results suggest that FABP4 contributes to obesity and associated metabolic disorders *via* its effects on fatty acid metabolism. As an adipokine, FABP4 has been reported to stimulate hepatic glucose production (95) and augment insulin secretion (96, 97), contributing to insulin resistance and hyperglycemia.

In addition to regulating lipid metabolism, recent studies show that FABP4 could also promote immune responses and inflammation in AT. In macrophages, FABP4 deficiency reduces pro-inflammatory cytokines *via* the attenuation of IKK-β/NF-κB activity (98). Moreover, FABP4-deficient macrophages show increased intracellular levels of the unsaturated fatty acid palmitoleate, which increases UCP2 expression *via* PPARγ and subsequently reduces mitochondrial oxidative stress and inflammasome activation (99, 100). Additionally, the loss of FABP4 in macrophages induces SIRT3 expression, which is also induced by monounsaturated fatty acids. Further, the loss of SIRT3 reverses the anti-inflammatory phenotype of FABP4 deficiency in macrophages (101). Recombinant FABP4 promotes inflammatory cytokine expression in 3T3-L1 adipocytes and in the AT of C57BL/6J mice *via* the p38/NF-κB pathway (94), further supporting its pro-inflammatory role in obesity-associated AT inflammation. Since FABP4 plays a critical role in coordinating cellular metabolism and inflammatory responses, numerous efforts have been taken to develop FABP4 inhibitors to treat immunometabolic diseases such as obesity, diabetes, and atherosclerosis. So far, there are hundreds of FABP4 inhibitors have been synthesized, offering promising hope for effective treatment strategies (102).

#### (8) PAI-1

Plasminogen activator inhibitor 1 (PAI-1; also known as serpin family E member 1 [SERPINE]) is a glycoprotein, 45 kDa in size, and belongs to the serine proteinase inhibitor (serpin) superfamily. PAI-1 is broadly expressed by various cells, including endothelial cells, adipocytes, hepatocytes, macrophages, platelets, and smooth muscle cells (103). PAI-1 has been recognized as the primary regulator of fibrinolysis owing to its quick inhibition on tissue plasminogen activator (tPA) and urokinase-type plasminogen activator (uPA) (104). Increased circulating PAI-1 levels and activities are critical factors contributing to increased cardiovascular risk in obesity 105). Moreover, circulating PAI-1 levels are also positively associated with obesity, insulin resistance, and metabolic syndrome (106). AT is a prominent source of PAI-1 in obesity (107, 108), and preadipocytes, adipocytes, and macrophages can produce PAI-1 and contribute to increased circulating PAI-1 levels in obesity (109, 110).

In addition to its classical role in regulating fibrinolysis, PAI-1 has also been implicated in the progression of obesity and associated metabolic dysfunction. PAI-1 expression is dramatically up-regulated in AT in humans with obesity and mice with HFD-induced obesity (111, 112). PAI-1 deficiency and PAI-1 inhibitor treatment can protect against HFD-induced obesity, insulin resistance, and liver steatosis (113–115), partially *via* the enhanced energy expenditure associated with alleviated hypothalamic leptin resistance (116) and increased adipocyte lipolysis (114, 117). The pro-inflammatory cytokines TNF-α and IL-6 stimulate PAI-1 expression in human AT and adipocytes *via* NF-κB activation, suggesting a close relationship between PAI-1 and obesity-induced inflammation (118, 119). Indeed, obese mice treated with PAI-1 inhibitor show a significant reduction in macrophage infiltration into white AT and increased anti-inflammatory M2 polarization (120). Further studies are required to elucidate the molecular mechanisms through which PAI-1 influences macrophage polarization. However, the current evidence suggests that targeting PAI-1 could provide a therapeutic strategy for obesity and metabolic syndrome.

#### (9) Follistatin-Like 1

Follistatin-like 1 (FSTL1) is a novel pro-inflammatory cytokine that is ~37 kDa in size and is highly expressed in AT, and mainly in the stromal vascular fraction 121). Moreover, FSTL1 expression is high in preadipocytes with a transient upregulation in early differentiated cells and then declines to basal levels after differentiation (121–123). Hence, FSTL1 has a potential role in adipogenesis. Indeed, a recent study found that FSTL1 deficiency inhibited preadipocyte differentiation *in vitro* and obesity development *in vivo* (123). In addition, FSTL1 has been shown to act as a novel stimulator of β-adrenergic signaling and promote BAT thermogenesis (124).

Recent studies have indicated a close relationship between FSTL1 and obesity-induced chronic inflammation and insulin resistance. Serum FSTL1 levels are higher in subjects with overweight, obesity, or T2DM than in healthy lean controls (125, 126). However, FSTL1 serum levels are found to be significantly lower in patients with severe obesity, which could be because of the reduction in adipogenesis and senescence of preadipocytes (127). In addition, FSTL1 expression is markedly increased in the AT of obese and T2DM mouse models (122). Although FSTL1 expression is low in adipocytes and macrophages, it can be induced by inflammatory stimuli such as TNF-α and LPS (121, 122), suggesting its potential role in mediating obesity-induced inflammation. Indeed, recombinant FSTL1 induces the expression of inflammatory cytokines (e.g., TNF-α, IL-6, and MCP-1) in both adipocytes and macrophages and impairs insulin signaling in adipocytes. Mechanistically, FSTL1 binds to CD14 and activates the inflammatory response *via* the TLR4-mediated activation of the IKKβ-NF-κB and JNK signaling pathways (122, 128). In addition, FSTL-1 is transported to the mitochondria, where it enhances ATP production and then activates the NLRP3 inflammasome, promoting IL-1β secretion from monocytes/macrophages (129). Together, these results suggest that FSTL1 could be a novel pro-inflammatory adipokine mediating AT inflammation in obesity. Nevertheless, further studies are required to elucidate its role in the development of inflammation and insulin resistance *in vivo*.

#### (10) MCP-1

Monocyte chemoattractant protein-1 (MCP-1 or CCL2), is a 11-kDa chemokine that recruits circulating monocytes and T lymphocytes into tissue by binding to its receptor, CC-chemokine receptor-2 (CCR2). Within AT, MCP-1 is produced by both adipocytes and stromal vascular fractions (4, 130) and is markedly elevated in the serum and AT in mouse models of obesity and individuals with obesity 131, 132). Several factors have been reported to upregulate MCP-1 expression in AT, including insulin (133) and hypoxia (134). Moreover, a recent study reported that the synergistic effect of elevated FFAs and TNF-α triggers MCP-1 production in monocytic cells *via* the TLR4/TRIF/IRF3 signaling cascade (135).

Many studies have demonstrated the critical role of MCP-1 in macrophage infiltration in AT during obesity and its contributes to the pathophysiology of obesity-associated inflammation and insulin resistance (136). For example, MCP-1 deficiency reduces macrophage infiltration in AT and improves metabolic profiles in *db/db* and HFD-induced obese models. In contrast, the overexpression of MCP-1 promotes macrophage infiltration into AT and results in an adverse metabolic phenotype (4). Similarly, the inhibition of CCR2 also attenuates diet-induced insulin resistance and hepatic steatosis in obese mice (137, 138), suggesting that MCP1/CCR2 could be a potential therapeutic target in obesity-associated metabolic complications. In addition, MCP-1 may also affect macrophage polarization, as MCP-1 deficiency increases M2 polarization to enhance WAT browning and increases energy expenditure (139). This suggests that targeting MCP1 could be a useful weight loss strategy.

Similar to AT, skeletal muscles also show increased macrophage infiltration, inflammation, impaired insulin signaling and systemic glucose tolerance in conditions of obesity (140). Moreover, the muscle-specific overexpression of MCP-1 induces macrophage accumulation in skeletal muscles and reduces muscle insulin sensitivity (140), suggesting that MCP-1 also involved in the etiology of T2DM. Notably, *in vitro* MCP-1 treatment impairs insulin signaling in skeletal muscle cells *via* the activation of ERK1/2 signaling (141). In addition, the acute infusion of recombinant MCP-1 in mice induces systemic insulin resistance without causing macrophage infiltration into AT (142), indicating that MCP-1 can also act as a potent inducer of insulin resistance in addition to its role in AT inflammation. The blockade of MCP-1/CCR2 signaling for inhibiting pro-inflammatory macrophage infiltration could be a promising therapeutic approach for obesity and diabetes (143, 144).

#### (11) SPARC

Secreted protein acidic and rich in cysteine (SPARC), also known as osteonectin or basement-membrane protein 40 (BM-40), is a 32-kDa matricellular glycoprotein that is highly expressed by AT and especially adipocytes (145–147). As a multifunctional ECM binding protein, SPARC plays important roles in cellular adhesion, tissue remodeling, and fibrosis (148).

SPARC expression is markedly up-regulated in AT in obese rodent models and humans with obesity (145, 147, 149), and is positively correlated with metabolic parameters such as fasting insulin and glucose levels, homeostatic model assessment for insulin resistance (HOMA-IR), waist circumference and hsCRP (147). Insulin and leptin have been shown to simulate SPARC expression in AT (147). Moreover, serum SPARC levels are also known to be elevated in individuals with obesity and T2DM and are positively correlated with BMI and insulin resistance (150, 151). Further, serum SPARC levels become reduced after bariatric surgery (152). Hence, adipose-secreted SPARC could have a role in obesity and diabetes.

SPARC knockout mice show increased white AT mass and adiposity after HFD consumption (153, 154), suggesting that SPARC limits AT expansion during obesity. This could be due to its role in collagen assembly or in inhibiting of the mitotic clonal expansion of preadipocytes at the early stage of adipogenesis *via* α5/β1 integrin and β-catenin signaling (155). Consistent with the increased expansion of AT, SPARC knockout mice also show lower inflammatory cell infiltration and IL-6 mRNA levels in both AT and the liver (156). Moreover, the overexpression of SPARC in 3T3-L1 adipocytes increases the levels of the pro-inflammatory cytokines IL-6, MCP1, and TNF-α and reduces those of the anti-inflammatory cytokine IL-10 (149). This suggests that SPARC promotes AT inflammation in obesity. Further studies are required to gain mechanistic insights into the effects of SPARC.

#### (12) SPARCL1

Secreted protein acidic and rich in cysteine-like 1 (SPARCL1) is a 75-kDa extracellular matrix protein belonging to the SPARC family. It is highly expressed in AT and mature adipocytes (157, 158). Like SPARC, SPARCL1 also mediates cell—matrix interactions and regulates many physiological processes, including cell adhesion, migration, proliferation, and differentiation (159). Although SPARCL1 has been intensively studied in the context of cancer, its role in AT and metabolic diseases has been reported only recently.

Using *in vitro* cellular differentiation models, several studies have demonstrated that SPARCL1 could be a negative regulator of adipogenesis. For example, Meissburger et al. compared the stromal vascular fraction (SVF) secretome between epididymal and inguinal AT (160). They identified SPARCL1 as a new protein factor preferentially secreted from epididymal SVF and found that it can inhibit adipocyte differentiation and may contribute to increased adipocyte hypertrophy in obesity. Consistent with this finding, Xiao et al. reported that SPARCL1 expression was higher in mature adipocytes than in early differentiating cells, and SPARCL1 interfered with preadipocyte differentiation *via* the Wnt/β-catenin signaling pathway (157). This indicated that SPARCL1 may act as a paracrine/autocrine factor to regulate adipogenesis. Further studies are required to unravel the role of SPARCL1 in regulating adipogenesis *in vivo.* Nonetheless, a recent study revealed the pathological role of SPARCL1 in the development of non-alcoholic steatohepatitis (NASH) in diet-induced mouse models (158). SPARCL1 mRNA levels were highly upregulated in white AT in NASH mouse models, while no changes were detected in mouse models of non-alcoholic liver disease (NAFLD). Moreover, the plasma levels of SPARCL1 were also higher in patients with NASH and were positively associated with parameters of liver injury. Moreover, the upregulation of SPARCL1, by either using recombinant SPARCL1 injection or SPARCL1 overexpression, accelerated hepatic inflammation and liver injury in mice with liver steatosis. In contrast, NASH pathogenesis was suppressed in mice with a SPARCL1 deficiency or SPARCL1 silencing in white AT as well as in those treated with a SPARCL1 neutralizing antibody. In addition, SPARCL1 treatment also promoted AT inflammation and impaired glucose tolerance, suggesting that SPARCL1 could be a mediator for the cross-talk between white AT and the liver in metabolic disorders. Mechanistically, SPARCL1 induces liver inflammation, at least in part, through the TLR4/NF-κB-dependent activation of MCP1. Thus, AT SPARCL1 could be targeted to treat obesity-associated NASH. More clinical studies are needed to further explore its relationship with obesity and associated metabolic disorders.

#### (13) SAA

Acute-phase serum amyloid A (SAA) is a family of evolutionarily conserved acute-phase proteins involved in host defense and acute inflammation. There are four isoforms of SAA. In humans, two SAA isoforms (SAA1 and SAA2) are primarily expressed in AT and adipocytes, whereas in mice, two homologs (SAA1.1 and SAA2.1) with more than 90% amino acid similarity to human SAA1 and SAA2 are mainly expressed in the liver 161). Moreover, mice express a third SAA isoform, SAA3, which is not expressed in humans (162). The SAA4 isoform is constitutively expressed at relatively low levels in both the human and mouse liver, although its function is unknown. Unlike SAA4, the expression of SAA1, SAA2, and SAA3 is highly induced in the AT and liver after inflammatory stimuli such as LPS treatment (163, 164).

Serum SAAs are elevated in obesity and reduce after weight loss, suggesting a close relationship between SAA levels and obesity (161, 165, 166). *In vitro* SAA treatment markedly increases the level of inflammatory cytokine (e.g., IL-6, IL-8, TNF-α, and MCP-1) production in macrophages, suggesting that SAA is a potent pro-inflammatory adipokine 161). In addition, *ex vivo* treatment with SAA can increase basal lipolysis in human AT 161), suggesting that SAA could exert a local lipolytic effect in AT. Mechanistically, SAA acts as an endogenous ligand for TLR4 and TLR2 (167, 168) and thereby activates inflammatory responses.

*In vivo* studies on SAAs have shown mixed results. Silencing SAA1.1 and SAA2.1 expression by antisense oligonucleotides prevented HFD-induced AT expansion and inflammation and improved glucose tolerance and insulin sensitivity in Swiss Webster mice (169). This suggested that SAA1.1 and SAA2.1 promote diet-induced obesity and inflammation. Similarly, SAA3 is also highly inducible in AT under conditions of obesity, and mice with an SAA3 deficiency are protected from HFD-induced obesity (170). Since humans do not express SAA3, SAA1 and SAA2 may be the major isoforms contributing to inflammation in human obesity. More recently, a study by Ji et al. generated mice lacking all three SAA isoforms (SAA1.1, SAA2.1, and SAA3) and reported no effect on obesity and associated complications (171). They observed higher levels of SAA isoforms in the AT, liver, and circulation of mice fed with an obesogenic diet. However, SAA isoform deficiency did not affect the development of obesity and AT inflammation (171). Thus, elevated SAA levels in obesity appear to be a consequence rather than a cause of AT inflammation. Consistent with this finding, AT-specific human SAA1(+/-) transgenic mice also showed no differences in HFD-induced obesity, insulin resistance, and obesity-related inflammation (172), ruling out the possible role of SAA1 in human obesity and associated inflammation. The discrepancies among different mouse studies could be due to the differences in mouse strains used or the use of mice lacking all three SAA isoforms versus mice with an SAA3 or SAA1.1/2.1 deficiency. Further studies are required to clarify the potential functional differences between the SAA isoforms in the context of obesity and the associated chronic inflammation.

### 2.2 Anti-Inflammatory Adipokines

#### (1) Adiponectin

Adiponectin is an AT-secreted protein with a molecular weight of ~30-kDa in mice and 28-kDa in humans (173, 174). As the most abundant adipokine in circulation, adiponectin is primarily secreted from the adipocytes of white AT and is expressed at low levels in other tissues, such as the liver and placenta (175). There are three forms of adiponectin in the plasma: low-molecular-weight (LMW), middle-molecular- weight (MMW), and high-molecular-weight (HMW) adiponectin. Compared with leptin and other pro-inflammatory cytokines, adiponectin shows an opposite trend in conditions of obesity. Adiponectin production in AT and serum levels of adiponectin are significantly reduced in obesity and are up-regulated after weight loss (176–178). Adiponectin targets several tissues, including the liver, skeletal muscle, and brain, indicating its role as a critical mediator for the crosstalk between AT and various metabolic organs (179.

Adiponectin functions as an insulin sensitizer and plays important roles in the regulation of glucose and lipid metabolism. For example, it suppresses hepatic gluconeogenesis and promotes glucose uptake and fatty acid β-oxidation in skeletal muscle (180). The overexpression of adiponectin increases AT expansion and improves insulin sensitivity in *ob/ob* mice and HFD-induced mouse models of obesity (181, 182). HMW adiponectin shows more prominent metabolic benefits (183). Adiponectin exerts its pleotropic actions *via* its two major receptors, AdipoR1 and AdipoR2. The binding of adiponectin to AdipoR1 promotes the activation of AMPK, while AdipoR2 activates the PPARα-mediated pathway (184). Moreover, APPL1 has been identified as an adaptor protein that links AdipoR1/2 to downstream signaling proteins. For example, APPL1 forms a complex with IRS1/2 and binds to insulin receptors to enhance insulin signaling *via* the PI3K-AKT pathway (185). Additionally, APPL1 mediates adiponectin-activated AMPK signaling by directly interacting with adiponectin receptors, and enhances LKB1 cytosolic translocation in muscle cells (186). Further, it has been demonstrated that adiponectin receptors have ceramidase activity and convert harmful metabolic ceramides into beneficial sphingosines to improve insulin sensitivity independent of AMPK (187, 188). These findings suggest that adiponectin is a promising adipokine that can improve insulin sensitivity and maintain energy homeostasis in the whole body.

In addition to its metabolic functions, adiponectin also exerts anti-inflammatory effects especially in cardiometabolic diseases. Obesity is associated with increased pro-inflammatory cytokine production in AT and the circulation (189). Serum adiponectin levels are inversely correlated with obesity (190) and inflammatory cytokine levels in T2DM (191). Pro-inflammatory cytokines reduce adiponectin expression in adipocytes (192). The *In vitro* treatment of macrophages with adiponectin inhibits the pro-inflammatory cytokine TNF-α and induces the expression of the anti-inflammatory cytokine IL-10 (193), possibly through the antagonism of TLR-mediated inflammatory responses (194). Furthermore, adiponectin promotes the removal of apoptotic debris (195). Since adipocyte apoptosis is a key initial contributor to macrophage infiltration into AT (196), the inhibition of adipocyte apoptosis using adiponectin may have therapeutic potential in treating obesity-associated metabolic disorders. In addition, several studies have reported that adiponectin induces the M1 to M2 macrophage polarization switch, thereby attenuating chronic inflammation (197–199). Mechanistically, adiponectin can regulate the histone demethylase JMJD3-IRF4 axis to drive the differentiation of M2 macrophages (200). In turn, macrophage polarization regulates AdipoR expression because M1 macrophages suppress AdipoR expression while M2 macrophages preserve it. Consequently, adiponectin induces pro-inflammatory responses in M1 macrophages while promoting IL-10 (anti-inflammatory cytokine) release in M2 macrophages (201). This suggests that macrophage polarization is an important determinant of the anti-inflammatory effect of adiponectin. Future studies are required to test this phenomenon in conditions of obesity to better understand the anti-inflammatory role of adiponectin in the context of obesity.

#### (2) Omentin

Omentin-1 is the main form of omentin in human blood (202). It is a newly identified adipokine that is preferentially expressed in visceral omental AT. It has insulin-sensitizing effects (203), and its production is downregulated in obesity (204). A lower level of plasma Omentin-1 contributes to the pathogenesis of obesity. The protective role of Omentin-1 against AT inflammation in obesity was investigated recently in FABP4-Cre-mediated Omentin-1 overexpression mice (205). The overexpression of Omentin-1 in AT prevented HFD-induced obesity and glucose intolerance, suppressed macrophage infiltration, and reduced pro-inflammatory cytokines in both AT and serum (205). Furthermore, Omentin-1 reduced thioredoxin-interacting protein (TXNIP)/NLR family pyrin domain containing 3 (NLRP3) signaling in the AT of obese mice and LPS-stimulated macrophages (205), suggesting that it suppresses AT inflammation in obese mice, at least partly, by inhibiting the TXNIP/NLRP3 signaling pathway. Since FABP4 is expressed in various cells in AT including adipocytes, preadipocytes, and macrophages, further studies are needed to use adipocyte- or macrophage-specific Cre mice to delineate the role of Omentin-1 in AT inflammation in obesity. In addition, Omentin-1 has recently been shown to attenuate LPS-induced oxidative stress and inflammation in macrophages by inhibiting the TLR4/MyD88/NF-κB signaling pathway (206). These studies suggest that Omentin-1 could reduce obesity-induced inflammation and therefore improve metabolic health in patients with obesity and associated metabolic disorders.

#### (3) ZAG

Zinc-alpha 2-glycoprotein (ZAG), first identified as an adipokine in 2004, mainly functions as a lipid mobilizing factor (207, 208). It is a 40-kDa soluble protein with a class I MHC protein fold (209). The expression of ZAG is significantly reduced in the serum and AT of patients with obesity and diabetes, and is negatively correlated with insulin resistance (210). *In vitro* ZAG treatment stimulates glycerol release from isolated murine adipocytes, and *in vivo* ZAG injection reduces body weight in *ob/ob* and *db/db* mice (211, 212). ZAG also binds to the β3-adrenergic receptor (β3-AR) and activates the cyclic adenosine monophosphate (cAMP)/PKA pathway to induce lipolysis in adipocytes (212).

In one study, ZAG expression was markedly reduced by the macrophage-secreted pro-inflammatory cytokine TNF-α (213). In addition, ZAG promoted white AT browning by inducing UCP-1 expression in white adipocytes (214–217. Furthermore, mice with hepatic ZAG overexpression showed attenuations in the LPS-induced inflammatory response and dyslipidemia in the serum as well as reduced liver steatosis (218). In contrast, ZAG knockout mice showed aggravated inflammatory responses, increased hepatic lipogenesis, impaired mitochondrial function, and increased β3-AR/PKA/adenosine monophosphate-response element binding protein (CREB) signaling (218). Mechanistically, ZAG overexpression increases β3-AR and PKA expression, promotes the formation of the CREB and CREB-binding protein (CBP) complex, and suppresses NF-κB-CBP complex formation, eventually alleviating inflammation in the liver. These results suggest that ZAG could be a potential anti-inflammatory adipokine that alleviates inflammation-induced liver steatosis in obesity. Nevertheless, more studies are required to test the anti-inflammatory effect of ZAG in AT in conditions of obesity.

#### (4) SFRP5

Secreted frizzled-related protein 5 (Sfrp5) was discovered in 2010 as a protein secreted by adipocytes. Sfrp5 was previously linked to the Wnt signaling pathway because it was shown to bind to and antagonize both Wnt5a and Wnt11 (219). Canonical Wnt signaling was found to negatively regulate adipogenesis (220). Sfrp5 expression was reduced in AT in mouse models of obesity and T2DM (221). Mice with Sfrp5 deficiency showed no significant differences in body weight, glucose disposal, or insulin sensitivity with a normal chow diet (221). However, a HFD lead to severe glucose intolerance and hepatic steatosis, as well as the accumulation of pro-inflammatory macrophages in AT in Sfrp5-deficient mice (221). These effects were associated with activation of the JNK1 signaling pathway, suggesting that Sfrp5 could play an anti-inflammatory role in obesity-induced inflammation and metabolic dysfunction. Consistent with these findings, the acute delivery of Sfrp5 adenovirus to *ob/ob* obese mice reduced AT inflammation and improved metabolic function (221). Mechanistically, Sfrp5 neutralizes non-canonical JNK1 activation by Wnt5a in macrophages and adipocytes *via* paracrine and autocrine mechanisms, respectively (221). Together, these data suggest that the Sfrp5-JNK1 axis in AT could be a potential target for the control of obesity-induced inflammation and metabolic abnormalities.

#### (5) CTRP3

C1q/TNF-related proteins (CTRPs) are a family of adiponectin paralogs that share common structural characteristics with C1q complement components and TNF receptor ligands (222). Currently, the CTRP family includes 15 members, of which CTRP1, CTRP2, CTRP3, CTRP5 and CTRP7 are predominantly expressed in AT (223). CTRP3 is the most well-studied CTRP in the context of metabolic disease and inflammation (224, 225).

CTRP3 is highly expressed in preadipocytes and shows a marked decrease in differentiated adipocytes (226, 227). Its expression is upregulated by insulin and downregulated by chronic LPS exposure in human adipocytes (227). Serum CTRP3 levels are also reduced in obesity (228). In addition to regulating hepatic glucose and lipid metabolism 229), CTRP3 also modulates adipocyte lipid metabolism in adipocytes. *In vitro* treatment with recombinant CTRP3 suppresses lipid accumulation in 3T3-L1 adipocytes and also inhibits adipocyte differentiation-related genes, such as PPARγ, C/EBPα, adiponectin, and FABP4 (226). These results suggest that CTRP3 may negatively regulate lipid metabolism during adipocyte differentiation. Whole-body CTPR3 knockout mice on a HFD show a reduced epididymal white AT weight and a lower expression of genes involved in lipogenesis, lipolysis, and adipogenesis (Maeda, T,2020). However, in adipocyte-specific CTRP3 knockout mice, neither adipocyte differentiation nor circulating CTRP3 concentrations are affected (230), indicating that compensatory mechanisms may be involved in rescuing the normal adipocyte phenotype.

Although its role in adipogenesis remains controversial, some studies indicate that CTRP3 participates in innate immune signaling in AT. For example, CTRP3 can act as an endogenous LPS antagonist in AT and as an inhibitor of TLR4-mediated chemokine and cytokine release in adipocytes and monocytes (231, 232). Mice receiving intraperitoneal injections of recombinant CTRP3 show attenuated LPS-induced inflammation in both AT and circulation (233), indicating that CTRP3 acts as a potent anti-inflammatory adipokine *in vivo*. Moreover, a recent study demonstrated that CTRP3 inhibits NOD1-mediated pro-inflammatory cytokine release from both adipocytes and THP-1 monocytes and attenuates LPS-induced NOD1 gene expression in murine AT *in vivo* (234). Detailed mechanistic studies are needed in the future to clarify the pathways by which CTRP3 mediates NOD1 activation in the AT under conditions of obesity. Overall, CTRP3 is a promising target for the treatment of obesity-associated chronic low-grade inflammation.

#### (6) Lipocalin 2

Lipocalin-2 (LCN-2) is a 25-kDa secretory glycoprotein, also called neutrophil gelatinase-associated lipocalin (NGAL), and was originally identified in mouse kidney cells and human neutrophil granules. In addition to neutrophils, LCN2 is selectively expressed in epididymal white AT and its expression is highly upregulated during 3T3-L1 adipocyte differentiation in a CCAAT/enhancer-binding protein (C/EBP)-dependent manner (235, 236). As a transporter of small and hydrophobic molecules such as steroids, fatty acids, retinol, prostaglandins, and hormones, LCN2 has been found to play a role in several processes, including hematopoietic cell apoptosis and innate immunity (237). Recent studies have suggested that LCN2 is secreted by adipocytes in response to inflammation. Further, it acts as an adipokine exerting protective effects against AT metabolic dysfunction in age-related obesity (238, 239).

Circulating LCN2 levels have been proposed as an inflammatory biomarker for obesity and its associated metabolic diseases (240). Serum LCN2 levels are higher in *ob/ob*, *db/db*, and HFD-induced obesity models and in human subjects with obesity. They are positively correlated with adiposity, hyperglycemia, insulin resistance, and the levels of pro-inflammatory cytokines and are reduced by thiazolidinedione (TZD) treatment (241–243). Although LCN2 mRNA expression is selectively upregulated in the AT and liver of obese animals (235, 236, 241), its protein expression is reduced in AT and is undetectable in the livers of obese mice (243, 244). Moreover, a very recent study revealed that bones are the primary contributor to increased circulating LCN2 levels in HFD-induced obesity models is mainly contributed by bone, as this increase is lost following the deletion of LCN2 in osteoblasts (243).

Since the LCN2 promoter region contains binding sites for several transcription factors, including NF-κB, STAT1, STAT3, CREB, and C/EBPβ (245, 246), its expression can be induced by inflammatory cytokines and metabolic stresses. For example, pro-inflammatory stimuli (e.g., TNF-α, IL-1β, and LPS), metabolic hormones (e.g., insulin), and nutrients (e.g., high glucose, palmitate, and oleate) strongly induce LCN2 expression in adipocytes *via* the activation of NF-κB 247). Hence, this secretory protein may be closely associated with inflammatory responses and metabolic stresses. As in adipocytes, LCN2 expression and secretion is also induced by LPS treatment in macrophages, indicating that macrophages are another important source of LCN2 production in AT during obesity (Guo, Jin, and Chen 2014).

The function of LCN2 in AT inflammation has been investigated in both adipocytes and macrophages. *In vitro* LCN2 treatment induces PPARγ expression and antagonizes TNFα-induced inflammation in adipocytes and suppresses LPS-stimulated cytokine expression in macrophages (236). Conversely, LCN2 knockdown decreases PPARγ and adiponectin expression in 3T3-L1 adipocytes. This suggests that the anti-inflammatory function of LCN2 is associated with its modulation of PPARγ activity (248). More recently, LCN2 was reported to play an anti-inflammatory role in regulating macrophage polarization by controlling the activation of the NF-κB-STAT3 loop 249). In conditions of LCN2 deficiency, bone marrow-derived macrophages (BMDM) tend to undergo M1 polarization and the activation of the NF-κB/STAT3 pathway is potentiated in response to LPS. In contrast, recombinant LCN2 inhibits LPS-stimulated M1 activation and NF-κB-STAT3 phosphorylation. Furthermore, under a HFD, LCN2 knockout mice show the enhanced expression of inflammatory cytokines such as MCP-1 and TNF-α in AT, along with reduced levels of anti-inflammatory Arg1 and PPARγ expression (244). Given the anti-inflammatory role of LCN2, elevated circulating LCN2 levels could be a protective mechanism against obesity-induced inflammation.

#### (7) Vaspin

Vaspin (also known as SERPINA12) belongs to the serine protease inhibitor family and was identified as an adipokine secreted from visceral AT in the Otsuka Long-Evans Tokushima fatty (OLETF) rat model of obesity and T2DM (250). Within AT, mature adipocytes show a significantly greater expression of vaspin than stromal vascular cells (250). Moreover, vaspin is also highly expressed in the liver and skin and moderately expressed in the brain, heart, and spleen (251). Several physiological stimuli have been reported to influence vaspin expression and secretion in white AT. For example, Gonzales et al. reported that age, gender, and nutritional status affect vaspin expression in gonadal white AT and insulin sensitizers induce its expression (252). Moreover, cold temperatures are reported to be a potent stimulator of vaspin expression in beige AT (251).

Several studies have suggested that vaspin plays a beneficial role of in counteracting obesity, insulin resistance, and inflammation. Vaspin mRNA expression is barely detected in the AT of lean humans and rodents, whereas it is upregulated in individuals with obese and T2DM (250, 253). In addition, circulating vaspin levels are positively correlated to vaspin mRNA expression in AT and are elevated in patients with obesity and T2DM (254–256) and reduced after weight loss (257). Both mRNA and serum levels of vaspin are associated with parameters of obesity and impaired insulin sensitivity (258, 259). Vaspin treatment and the adipocyte-specific overexpression of vaspin ameliorate HFD-induced obesity, liver steatosis, and insulin resistance (250, 260, 261), suggesting that the elevation of vaspin levels could be a compensatory mechanism in obesity. Conversely, vaspin knockout mice with high fat-high sucrose diet demonstrate increased body weight, AT macrophage infiltration, hepatic lipid accumulation, and deterioration of insulin sensitivity (260). Although the role of vaspin in adipogenesis remains controversial, this adipokine has been shown to directly increase insulin sensitivity in 3T3-L1 adipocytes (262, 263) and hepatocytes (260, 264) by enhancing ATK signaling. In addition, a recent study showed that vaspin attenuates the IL-1β-induced pro-inflammatory cytokine response by inhibiting NF-κB signaling in adipocytes (263). Although the molecular mechanisms underlying vaspin action and signaling in AT remain unclear, several studies have highlighted its role as a ligand for the GPR78/MTJ-1 complex in the liver under conditions of ER stress and as a ligand for GPR78 and voltage-dependent anion channels (VDAC) in endothelial cells (265). Moreover, as a serine protease inhibitor, vaspin has been shown to inhibit the activity of the insulin-degrading serine protease kallikrein 7 (KLK7) in pancreatic islets (261), which could increase the duration of insulin in circulation. This physiological mechanism could also explain its compensatory effects on obesity-induced insulin resistance. Further studies are required to test these mechanisms in adipocytes.

#### (8) IL-10

Interleukin-10 (IL-10) is an anti-inflammatory cytokine produced by a variety of cells in the innate and adaptive immune system, — including macrophages, natural killer (NK) cells, T cells, and B cells— in response to pro-inflammatory stimuli. IL-10 binds to the high affinity α chain of its receptor (IL-10Rα) and oligomerizes with the IL-10Rβ subunit, activating the downstream JAK/STAT3 signaling pathway to exert its anti-inflammatory function. In AT, IL-10 is mainly produced by macrophages (266, 267). Moreover, IL-10 expression in haematopoietic cells induces IL-10 expression in the AT of HFD-fed obese mice (268), further suggesting that the IL-10 in AT does not come from bone marrow but resident cells.

As an anti-inflammatory cytokine, IL-10 has been suggested to attenuate AT inflammation and improve insulin sensitivity in conditions of obesity. Serum IL-10 levels are significantly reduced in patients with obesity and metabolic syndromes such as hypertriglycemia (269, 270). Similarly, IL-10 and STAT3 mRNA expression levels are also reduced in the AT of HFD-fed obese rats (270). In contrast, a recent study reported that IL-10 was upregulated in subcutaneous white AT in female patients with obesity and insulin resistance (267). Nonetheless, increasing IL-10 levels *via* transgenic overexpression or the administration of recombinant IL-10 decreases inflammation and improves insulin sensitivity (271–273). Mechanistically, IL-10 primarily exerts its anti-inflammatory action in AT by modulating macrophage activation. This is further evinced by the observation that IL-10 does not affect metabolic functions in human adipocytes owing to the low expression of IL-10Rα in adipocytes (267). *In vitro* IL-10 treatment suppresses lipid-induced TNF-α production in Raw264.7 macrophages (268). Moreover, *in vivo* IL-10 treatment promotes M2 macrophage polarization in the epididymal white AT of obese mice (266, 274) and therefore attenuates obesity-induced inflammation. Notably, the liposome-mediated delivery of IL-10 into macrophages results in significant anti-obesity and anti-inflammatory effects, including a marked reduction in epididymal white AT, liver steatosis, and AT inflammation in HFD-fed mice (275). Additionally, IL-10 shows some structural similarities to leptin, which can ameliorate hyperphagia and suppress hypothalamic inflammation in obese mice (276, 277). Thus, targeting AT IL-10 could be a potential therapeutic strategy for obesity and its associated metabolic disorders.

#### (9) IL-1RA

IL-1 receptor antagonist (IL-1RA) is a member of the interleukin (IL)-1 family that binds to IL-1 receptors and antagonizes the pro-inflammatory cytokines IL-1α and IL-1β. IL-1RA is highly expressed in AT and the liver, and it is further upregulated in obesity (278). In line with this, serum IL-1RA levels are elevated in patients with obesity and are even higher in those with glucose intolerance and T2DM (279), likely to counteract the increase in the pro-inflammatory cytokine IL-1 in obesity. In contrast, serum IL-1RA levels significantly decline after weight loss through gastric bypass surgery (279, 280), indicating that white AT could be a major source of IL-1RA. In addition, a recent study reported a positive correlation between serum IL-1RA levels, and the expansion of subcutaneous AT and ectopic fat deposition in visceral AT, the liver, and the pancreas (281). This suggested that as an adipokine, IL-1RA could potentially explain the link between obesity and other metabolic dysfunctions. Further studies are required to test the causal relationship between IL-1RA levels and obesity and associated chronic inflammation.

In addition, IL-1RA has also been implicated in the control of energy homeostasis. IL-1RA knockout mice show a lean phenotype due to a defect in adipogenesis and increased energy expenditure (282). The increased energy expenditure is associated with the activation of the sympathetic nervous system, which produces catecholamine to activate brown AT and promotes the browning of white AT depots. Unsurprisingly, IL-1RA knockout mice are resistant to HFD-induced obesity and show enhanced insulin sensitivity (283) due to increased energy expenditure, suggesting that increased serum IL-1RA levels might contribute to the pathogenesis of obesity and associated metabolic abnormalities. Interestingly, the levels of circulating pro-inflammatory cytokines (e.g., IL-6, TNF-α, and IL-1β) are normal or even low in IL-1RA knockout mice, indicating that inflammatory changes in response to a HFD are unlikely to be responsible for the lean phenotype. Nonetheless, recombinant IL-1RA (Anakinra) has been found to be beneficial in animal models (284) of diabetes and patients with diabetes (285), suggesting that excess IL-1 signaling contributes to beta cell damage in diabetes (286). Further studies are required to evaluate whether targeting IL-1RA in AT could offer therapeutic benefits in patients with obesity.

## 3 Hepatokines

### 3.1 Fetuin A

Fetuin-A (FetA) is a fatty acid-binding glycoprotein that is abundant in serum and is primarily expressed in and secreted from the liver in adulthood (287). Accumulating evidence suggests that FetA is closely associated with metabolic syndrome and T2DM (288, 289). Serum FetA levels are higher in human subjects with impaired glucose tolerance and liver steatosis (290), suggesting that FetA production is increased in fatty livers, promoting insulin resistance and T2DM. Indeed, it has been shown that FetA is a natural inhibitor of insulin receptor tyrosine kinase phosphorylation and blocks insulin signaling transduction (291). Furthermore, FetA knockout mice show improved insulin sensitivity and resistance to weight gain (292). Moreover, the expression of FetA is strongly induce by saturated fatty acids in HepG2 cells *via* NF-κB activation (293). In turn, FetA acts as an endogenous ligand of TLR4 to promote lipid-induced insulin resistance in adipocytes (294).

FetA has also been implicated in AT inflammation in obesity. FetA promotes the secretion of pro-inflammatory cytokines from both adipocytes and macrophages (294, 295) while reducing adiponectin levels in human adipocytes (296). FetA acts as a carrier of FFA and presents FFA to TLR4 297). Meanwhile, it also binds to region of leucine-rich repeats (LRR) 2 and 6 in the TRL4 extracellular domain to induce pro-inflammatory cytokine release *via* the activation of the NF-κB pathway (294). FetA knockdown mice show decreased expression of pro-inflammatory cytokines such as IL-6 and TNF-α in adipocytes (294).

However, FetA also serves as a chemoattractant for macrophages, inducing the migration and infiltration of macrophages into AT (298). Moreover, FetA promotes the pro-inflammatory M1 polarization of macrophages (298). Mechanistically, FetA promotes M1 macrophage polarization *via* c-Jun and JNK- mediated IFNγ upregulation, which induces the expression of MCP-1 and iNOS in adipocytes *via* the JAK2-STAT1 pathway (299). Taken together, these studies show that FetA is an upstream regulator of FFA-induced TLR4 activation in adipocytes and promotes M1 macrophage polarization. Hence, FetA as a potential novel therapeutic target for obesity-induced inflammation and T2DM.

### 3.2 DPP4

Dipeptidyl peptidase 4 (DPP4), also known as T cell surface marker CD26, is a transmembrane glycoprotein sized ~110 kDa. It is expressed on the surface of many cells, including endothelial cells (300), immune cells (301), and adipocytes (302). Early studies suggested that DPP4 was an adipokine since it was shed from the adipocyte membrane *via* MMP9 and formed soluble DPP4 (sDPP4) in circulation (302, 303). However, DPP4 expression and activity are much higher in the liver than in adipose tissue (300) and recent studies have found increased DPP4 expression and secretion from hepatocytes in obese mice (304, 305), indicating its role as a novel hepatokine in mediating the complex crosstalk between hepatocytes and adipocytes. In line with these findings, mice with hepatocyte-specific DPP4 knockdown have shown a significant reduction in DPP4 activity in the serum along with reduced AT inflammation, insulin resistance, and glucose intolerance (305). Likewise, the selective loss of DPP4 in adipocytes has been found to increase hepatic insulin sensitivity and inflammation, although no effects on glucose tolerance have been seen (300, 306). This further supports the idea that hepatocyte-secreted DPP4 is a major contributor to increased serum DPP4 activity during obesity.

DPP4 plays a primary role in cleaving N-terminal dipeptides and its well-known substrates are incretin hormones such as glucagon-like peptide-1 (GLP-1) and gastric inhibitory polypeptide (GIP). By cleaving GLP-1 and GIP, DPP4 impairs insulin secretion from pancreatic islets and subsequently increases glucose levels in the circulation (307). Notably, of DPP4 inhibitors have been developed as novel drugs for T2DM because they prevent the enzymatic degradation of incretin peptides (308). In addition, DPP4 also plays a non-enzymatic role in regulating immune cells and the AT inflammation associated with obesity and diabetes. The overexpression of DPP4 in hepatocytes accelerates HFD-induced obesity and AT inflammation and liver steatosis (309). By contrast, the lack of DPP4 attenuates HFD-induced AT inflammation and insulin resistance and enhances adiponectin levels, despite increases in visceral fat mass (310). Similarly, DPP4 inhibition improves glucose tolerance, β-cell function, and AT inflammation in *db/db* mice (311). Mechanistically, DPP4 produces an inflammatory microenvironment in AT by activating T cells and promoting their proliferation *via* interaction with adenosine deaminase (ADA) in human DCs (301). Moreover, DPP4 inhibition reduces M1-polarized macrophage migration while inducing the M2 phenotype in AT and the liver *via* macrophage inflammatory protein-1α, thereby attenuating obesity-induced inflammation and insulin resistance (312). More importantly, DPP4 inhibitors exert a direct anti-inflammatory effect on both macrophages and adipocytes by suppressing LPS-induced NF-κB activation (313). Together, these studies suggest that targeting DPP4 could be effective for managing inflammation in obesity.

### 3.3 FGF21

Fibroblast growth factor-21 (FGF-21) is a ~22-kDa protein that belongs to the FGF family. Unlike conventional FGF proteins, FGF21 functions as an endocrine hormone with diverse metabolic functions. It is preferentially expressed in the liver (314), although its expression is also noted in the with pancreas, skeletal muscle, and AT (315) as well as in the hypothalamus of the brain (316). In the mouse liver, FGF21 levels are significantly elevated under fasting conditions and a ketogenic diet *via* PPARα regulation (317, 318). Therefore, FGF21 acts as a stress hormone and helps the body to cope with nutrient restriction. Paradoxically, both the circulating and tissue (e.g., liver, skeletal muscle, and AT) levels of FGF21 are elevated under nutrient-excess states such as obesity (319), suggesting the presence of FGF21 resistance (320) or an adaptive response in obesity.

As a novel metabolic regulator, FGF21 plays a critical role in promoting glucose uptake and lipid metabolism in obesity. It exerts strong anti-obesity and anti-diabetes effects by binding to its receptor FGFR1 and co-receptor β-Klotho (321–323). FGFR1 and β-Klotho are highly expressed in many metabolic tissues, especially AT (315, 323), suggesting that AT is one of the major targets of FGF21. The effect of FGF21 on glucose uptake in adipocytes is mediated by the increased expression of GLUT1 (324). Since skeletal muscles are major sites of circulating glucose utilization, it remains to be examined whether the adipose-mediated increase in glucose uptake can account for the glucose-lowering effect of FGF21; thus, further *in vivo* studies are required. Moreover, FGF21 stimulates the browning of white AT by increasing PGC1α-dependent UCP-1 expression (325), which potentially contributes to its metabolic benefits in obesity and diabetes. Given the promising therapeutic potential of FGF21 in metabolic diseases, gene therapy (321) and many FGF21 analogs have been developed. These strategies have shown promising results in treating dyslipidemia but not hyperglycemia (326), suggesting that a combination of FGF21 with other glucose-lowering agents could be an option for treating obesity-related metabolic complications.

Recently, several studies have suggested that FGF21 has an anti-inflammatory effects, strengthening the evidence supporting its metabolic benefits. For example, FGF21 blocks the nuclear translocation of NF-κB in adipocytes and AT under conditions of insulin resistance, ameliorating inflammation in diabetes and simultaneously improving glucose metabolism (327). Similarly, FGF21 can inhibit LPS-induced inflammation in macrophages *via* the Nrf2-dependent upregulation of HO-1 and suppression of NF-κB signaling (328). Moreover, FGF21 treatment can improve insulin sensitivity and induce adiponectin expression and release from adipocytes *via* PPARγ, and subsequently increase circulating adiponectin levels (329). FGF21 overexpression in the liver *via* hydrodynamic delivery protects against HFD-induced obesity and adipose inflammation and improves glucose homeostasis (330). Conversely, the pro-inflammatory cytokine TNF-α downregulates the levels of β-Klotho and induces inflammation, thus impairing the beneficial effects of FGF21 on obesity and ER stress (319). It has been shown that TNF-α acts through the TNF-α-JNK1 axis to inhibit the effects of FGF21 in AT, suggesting that a combination of FGF21 and JNK1 inhibitors could be a potential therapeutic strategy in obesity (319). In addition, FGF21 plays a key role in hypothalamic inflammation, and the decrease in FGF21 levels induces obesity-related hypothalamic inflammation (316). Moreover, FGF21 can also limit inflammation in the pancreas of HFD-induced mouse models of obesity (331). Overall, in addition to its metabolic effects, FGF21 could also act as an anti-inflammatory factor for the treatment of obesity-induced metabolic dysfunctions.

### 3.4 GDF15

Growth differentiation factor 15 (GDF-15), a ~25-kDa secreted homodimeric protein, is an atypical member of the TGFβ superfamily (332). It is broadly expressed at high levels in placenta, kidneys, liver, and intestine. Its circulating levels are markedly elevated during cellular stress conditions such as mitochondrial dysfunction (333).

Accumulating evidence has suggested that GDF-15 is a stress-responsive cytokine that is closely associated with metabolic disorders such as obesity and diabetes (334). Serum GDF15 levels are elevated in both rodent models and patients with obesity and T2DM and are positively associated with body weight and AT mass (335–337). GDF15 mRNA expression is also markedly up-regulated in the liver and AT of obese mice (337, 338). In patients with severe obesity, GDF15 expression is mostly restricted to the liver instead of AT and is reduced after bariatric surgery (339), suggesting that the liver could be a major source of GDF15 in obesity.

GDF15 knockout increases adiposity and food intake (340) whereas GFD15 overexpression reduces body weight, fat mass, and food intake, and improves insulin sensitivity under both a normal chow diet and HFD (341). This suggests that GDF15 is a regulator of body weight and appetite. Mechanistically, GDF15 binds to its high- affinity receptor glial cell-derived neurotrophic factor family receptor alpha-like (GFRAL), which is highly expressed in the hindbrain-brainstem region and suppresses food intake (342). In addition to inhibiting appetite, GFD15 also contributes to weight loss by increasing energy expenditure. For example, recombinant GDF15 treatment decreases body weight and improves insulin sensitivity by enhancing thermogenesis, lipolysis, and fatty acid oxidation in AT, the liver and muscles (333, 343). Moreover, in one study, an engineered long-acting recombinant GDF15 protein was shown to exert a strong weight-lowering effect in obese mice and monkeys (337), confirming its potential application for treating obesity and associated metabolic disorders.

In addition, the metabolic benefits of GDF15 could also be attributed to its anti-inflammatory properties. In lean mice, GDF15 overexpression can reduce inflammatory responses to LPS and body weight (344). Similarly, GDF15 transgenic mice fed a HFD show reduced NLRP3 inflammasome activity and pro-inflammatory macrophage infiltration into white AT as well as lower serum leptin and insulin levels (345), suggesting that GFD15 could reduce obesity-associated inflammation and improve insulin sensitivity. Moreover, a study found that recombinant GDF15 also reduces serum leptin and pro-inflammatory cytokine levels while increasing adiponectin levels in HFD-induced obese mouse models (346). Conversely, GDF15 monoclonal antibody treatment was found to accelerate HFD-induced adiposity, liver steatosis, and insulin resistance, and increases the expression of pro-inflammatory cytokines in AT (347). Notably, studies on GDF15 treatment often report changes in body weight and AT mass, which could contribute to the observed anti-inflammatory effect. Recently, a study reported the direct anti-inflammatory effect of GDF15 in macrophages. GDF15 expression was upregulated by the anti-inflammatory cytokine IL-4 and promoted M2-like macrophage polarization *via* the upregulation of oxidative metabolism (348). Collectively, these findings suggest that GDF15 may be a promising therapeutic target for the regulation of both energy metabolism and the AT immune microenvironment in metabolic diseases.

### 3.5 MANF

Mesencephalic astrocyte-derived neurotrophic Factor (MANF) is ~21-kDa protein that is preferentially expressed in tissues with high metabolic activity and secretory function, such as the brain, pancreas, and liver (349). Its expression and secretion are induced by several stress signals such as ER stress (350) and fasting (351). Moreover, dietary restriction also increases MANF levels in the liver and circulation (352), suggesting that MANF expression and secretion are highly regulated by nutritional status.

A recent study identified MANF as a hepatokine induced by feeding and revealed its potential role in energy homeostasis and obesity (353). Circulating MANF levels are elevated in human subjects with overweight and are positively correlated with BMI (353). The liver-specific overexpression of MANF reduces HFD-induced obesity by promoting inguinal white AT browning and thermogenesis. Furthermore, liver-specific MANF ablation impaires white AT browning and worsens obesity. This suggests that MANF is an anti-obesity hepatokine and that elevated serum levels of MANF could represent a compensatory effect occurring in response to overweight. Notably, the intravenous injection of recombinant MANF-Fc reduces obesity and related metabolic abnormalities by increasing thermogenesis in both HFD-induced and *ob/ob* obese mouse models. Mechanistically, MANF can directly promote the browning of white adipocytes *via* p38 MAPK signaling. Further studies are required to identify the receptor of MANF and to explore the mechanisms contributing to MANF elevations in patients with obesity.

In addition to regulating energy homeostasis, MANF also plays a beneficial role in attenuating inflammation and associated metabolic disorders. For example, MANF heterozygous mice exhibit inflammatory phenotypes in various tissues along with progressive liver damage, fibrosis, and steatosis (354). Conversely, mice with the liver-specific overexpression of MANF show decreased adipose inflammation, insulin resistance, and hepatic steatosis (353). One possible mechanism for effect of MANF could be its ability to bind to p65 through its C-terminal SAP-like domain, which could suppress NF-κB activation (355). Further studies are required to examine its anti-inflammatory effect in AT *in vivo*.

## 4 Myokines

### 4.1 Irisin

Irisin is a recently discovered exercise-induced myokine that is cleaved from fibronectin type III domain-containing protein 5 (FNDC5) in skeletal muscle (356). It is composed of 112 amino acids peptide, has a predicted molecular weight of 12 kDa, and shows high homology between humans and mice. In addition to skeletal muscle, irisin is also produced in AT (357). irisin was first reported to be a potent stimulator facilitating the conversion of white AT into beige AT and promoting thermogenesis by increasing UCP-1 expression (356). Subsequently, irisin was shown to promote browning and thermogenesis in mature human adipocytes derived from subcutaneous white AT *via* the p38 and ERK-mediated induction of UCP-1 expression (358). Moreover, αVβ5 integrin was identified as irisin receptor and was found to mediate its effects on thermogenesis (359). These findings suggest that irisn is a mediator of muscle—adipose crosstalk and has a great therapeutic potential for obesity treatment. Moreover, *in vitro* irisin treatment significantly inhibits the differentiation of human primary preadipocytes into mature adipocytes (360) while FDNC5 silencing in preadipocytes enhances adipogenesis (361). This indicates that irisin is a negative regulator of adipogenesis in obesity. In addition, it also exerts anti-obesity effects by stimulating basal lipolysis in adipocytes. Obese mice with lentiviral FNDC5 overexpression or irisin infusion show an amelioration of metabolic glucose and lipid dysregulation. This effect is mediated by enhanced basal lipolysis in adipocytes *via* the cAMP-PKA-hormone sensitive lipase (HSL)/perilipin pathway (362). In addition, recent studies have demonstrated the anti-inflammatory properties of irisin, showing that it can suppress the inflammatory responses in various cells — including adipocytes, macrophages, hepatocytes, pancreatic β cells, and endothelial cells — *via* TLR4/MyD88-mediated NF-κB and NLRP3 inflammasome pathways 363–366). This suggests that irisin has potential as a potent immunometabolic regulator for obesity and its associated metabolic comorbidities. Further studies are required to test its anti-inflammatory role *in vivo*.

Although some discrepancies has been reported, most studies have found that serum irisin levels are positively correlated of with obesity but negatively correlated with T2DM. Serum irisin levels are elevated in subjects with overweight or obesity and reduced after weight loss (367), while they are lower in T2DM (368). Hence, elevated irisin could act as a protective myo-adipokine against obesity and related metabolic disorders.

### 4.2 IL-6

Interleukin-6 (IL-6) is a ~24-kDa cytokine that belongs to the gp130 cytokine superfamily. Conventionally, IL-6 is classified as a pro-inflammatory cytokine and is produced by immune cells and other somatic cells at the site of inflammation. The pro-inflammatory role of IL-6 is mediated by trans-signaling, which occurs when IL-6 binds to the soluble form of the IL-6 receptor (sIL-6R) and gp130, but not to IL-6R, to activate JAK/STAT3 signaling in inflammatory cells (369). In obesity, circulating IL-6 levels are elevated and are associated with-low grade inflammation and dysregulated metabolism, as described previously (370). IL-6 is also one of the most important and abundant myokines released from skeletal muscle after exercise (371), and it has been shown to have anti-obesity and anti-inflammatory functions. For example, IL-6 deficiency increases adiposity and subcutaneous AT mass, and this increase which is partly reversed by IL-6 supplementation (372). Moreover, the exercise-induced reduction of visceral AT mass is abolished in patients with obesity when IL-6 signaling is blocked, suggesting that IL-6 is required for the exercise-mediated reduction of visceral AT mass (373). This could be attributed to the effect of IL-6 on lipid metabolism. Both *in vitro* and *in vivo* studies have shown that IL-6 stimulates lipolysis and fatty acid oxidation in adipocytes *via* the activation of AMPK (374, 375).

Moreover, acute exercise also upregulates IL-6 signaling (e.g., IL-6R, gp130, SOCS3, and STAT3 phosphorylation) in the AT of HFD-induced obese mouse models, and this is associated with a reduction in M1 macrophages and AT inflammation (376, 377), suggesting that IL-6 singling in adipose tissue could contribute to the beneficial effect of exercise on adipose tissue inflammation. Indeed, it has been reported that IL-6/STAT3 signaling promotes M2 macrophage polarization and proliferation by inducing IL-4R expression (378, 379). Conversely, mice with the myeloid cell-specific deletion of IL-6Rα show enhanced systemic inflammation and glucose intolerance 378), suggesting that IL-6 plays an unexpected role in limiting inflammation in obesity. Moreover, the selective blockade of IL-6 trans-signaling (and not IL-6R) prevents AT macrophage accumulation in HFD-induced obese mouse models without exacerbating insulin resistance (380), suggesting that targeting IL-6 trans signaling could also be a more favorable option for inflammatory diseases. Nevertheless, further studies are required to test its anti-inflammatory effect in human obesity.

### 4.3 DEL-1

Developmental endothelial locus-1 (DEL-1) is a 52-kDa glycoprotein secreted by vascular endothelial cells during embryological vascular development (381). It was previously shown that DEL-1 inhibits the adhesion of leukocytes to the endothelium and prevents the initiation of inflammation by blocking the interaction between LFA-1 (αLβ2) on leukocytes and ICAM-1 on the endothelial cells (382). Moreover, DEL-1 also reduces inflammation by enhancing the macrophage efferocytosis of apoptotic cells (383) after binding to the “eat-me” signal phosphatidylserine on apoptotic cells and the αvβ3 integrin on macrophages.

A recent study reported that DEL-1 could mediate the beneficial effects of exercise on obesity-associated inflammation and insulin resistance (384). DEL-1 mRNA expression was reduced in skeletal muscle from patients with obesity or diabetes, and this reduction was reversed by exercise in a time-dependent manner. Moreover, a study found that *in vitro* DEL-1 treatment ameliorates palmitate-induced inflammation and impairs insulin signaling in 3T3-L1 adipocytes *via* AMPK/HO-1 signaling (384). Similarly, DEL-1 also reduces LPS-stimulated NF-κB activation and pro-inflammatory cytokine secretion Raw264.7 macrophages (385). Moreover, DEL-1 acts locally to attenuate palmitate- and HFD-induced skeletal muscle ER stress and insulin resistance *via* SIRT1/SERCA2-mediated signaling (386). Together, these findings suggest that DEL-1 is an exercise-induced protein that reduces obesity-associated inflammation and insulin resistance. Further human studies and genetic *in vivo* animal studies are needed to verify the relationship of DEL-1 with human obesity and to unravel its role *in vivo*.

## 5 Potential Clinical Significance and Implications of Organokines

Obesity is characterized by low-grade chronic inflammation, which contributes to obesity-associated metabolic disorders. After conducting a literature search of the organokines that could play a role in AT inflammation, we reviewed 15 pro-inflammatory and 15 anti-inflammatory organokines (Supplementary Table 1). All the pro-inflammatory organokines show increased levels in patients with obesity, suggesting that they could play pathological roles in AT inflammation in obesity. Among them, pro-inflammatory organokines such as chemerin, progranulin, RBP4, FABP4, PAI-1, MCP-1, SPARC, SPARCL1, SAA, Fetuin A, and DPP4 have been shown to contribute to AT inflammation in pre-clinical animal models of obesity, suggesting that they could serve as potential therapeutic targets for obesity-induced inflammation. Similarly, among the 15 anti-inflammatory organokines, 7 (adiponectin, Omentin-1, ZAG, SFRP5, CTRP3, IL-10, and DEL-1) showed reduced levels and 8 (LCN2, VASPIN, IL-1RA, FGF21, GDF15, MANF, Irisin, and IL-6) showed elevated levels in patients with obesity. The reduction of anti-inflammatory organokines could also contribute to the development of AT inflammation in obesity, while the increase in anti-inflammatory organokines could represent an adaptive response to obesity.

## 6 Conclusions

AT inflammation is a hallmark of obesity and plays a critical role in the development of obesity-associated metabolic diseases. AT, the liver, and skeletal muscles are now considered endocrine organs as they produce bioactive molecules (termed adipokines, hepatokines, and myokines, respectively). These organokines have been shown to mediate the crosstalk between various tissue *via* endocrine, paracrine, and autocrine pathways and could act as novel targets for obesity-induced inflammation and insulin resistance.

**Figure 1:**
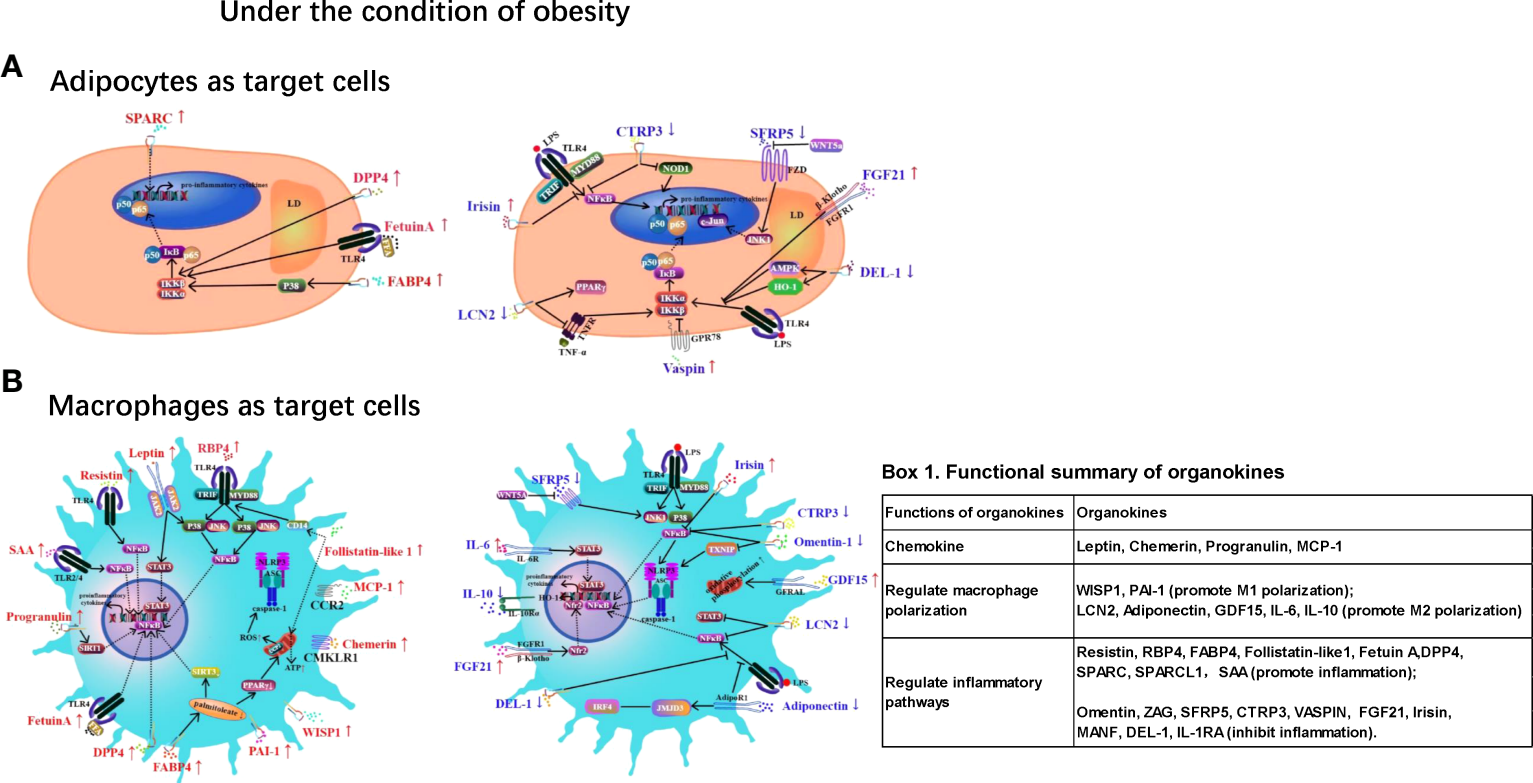
Summarized mechanisms of the organokines in regulation of adipose tissue inflammation under obesity condition. Adipocytes **(A)** and macrophages **(B)** are the primary target cells. Red colors symbols reflect pro-inflammatory organokines, blue symbols reflect anti-inflammatory organokines. Red upward arrows reflect an increase of the organokines in obesity and blue downward arrows reflect decreased level of organokines in obesity. The overall roles of organokines has been categorized into 3 groups: chemokine, regulation of macrophage polarization and regulation of inflammatory pathways (Fig 1, box 1). CTRP3: C1q/TNF-related proteins; DEL-1: Developmental endothelial locus-1; DPP4: Dipeptidyl peptidase 4; FABP4: Fatty acid binding protein 4; FFA: Free Fatty Acids; FGF21: Fibroblast growth factor-21; GDF15: Growth differentiation factor 15; LCN2: Lipocalin-2; MCP-1: Monocyte chemoattractant protein-1; PAI-1: Plasminogen activator inhibitor 1; RBP4: Retinol-binding protein-4; SAA: serum amyloid A; SFRP5: Secreted frizzled-related protein 5; SPARC: Secreted protein acidic and rich in cysteine; WISP1: Wingless‐type (Wnt)-inducible signaling pathway protein-1.

In this review, we summarized up to 30 organokines that have been shown to exert pro-inflammatory or anti-inflammatory effects in obesity and highlighted their roles and molecular effects in obesity and AT (**Supplementary Table 1**). Functionally, these organokines can be categorized into three groups: chemokines, molecules regulating macrophage polarization, and molecules regulating inflammatory pathways (**Figure 1**, box1). For example, leptin, progranulin, chemerin, and MCP-1 act as chemokines for immune cell (macrophages and DCs) infiltration into AT during obesity. WIPS1 and PAI-1 are involved in promoting M1 macrophage polarization, while LCN2, adiponectin, GDF15, IL-6, and IL-10 promote M2 macrophage polarization. Moreover, adipokines (e.g., resistin, RBP4, FABP4, Follistatin-like 1, SPARC, SPARCL1, and SAA) and hepatokines (Fetuin A and DPP4) are direct activators of inflammatory pathways such as the NF-κB, JNK, and NLRP3 inflammasome pathways in both adipocytes and macrophages. In contrast, organokines such as Omentin-1, ZAG, SFRP5, CTRP3, VASPIN, FGF21, irisin, MANF, DEL-1, and IL-1RA act as antagonists of inflammation. An improved understanding of the roles and molecular mechanisms of these organokines could greatly facilitate the translation of their regulatory effects on inflammation into the prevention and treatment of obesity-induced metabolic diseases.

## Author Contributions

Conceptualization: YR, DG, and HG; literature search: HZ, CY, XL, LW, and XD; writing: YR, HZ, and DG; review and editing: YR, DG, CY, XL, LW, XD, and HG. All authors contributed to the article and approved the submitted version.

## Funding

This work is supported the National Natural Science Foundation of China (NFSC) (Grant No. 81873665), China Postdoctoral Science Foundation (Grant No. 2018T111073, No.2017M613150) to DG.

## Conflict of Interest

The authors declare that the research was conducted in the absence of any commercial or financial relationships that could be construed as a potential conflict of interest.

## Publisher’s Note

All claims expressed in this article are solely those of the authors and do not necessarily represent those of their affiliated organizations, or those of the publisher, the editors and the reviewers. Any product that may be evaluated in this article, or claim that may be made by its manufacturer, is not guaranteed or endorsed by the publisher.
