# Supplementary material for: Adipokines, Hepatokines and Myokines: Focus on Their Role and Molecular Mechanisms in Adipose Tissue Inflammation
Source: Front Endocrinol (Lausanne). 2022 Jul 14;13:873699. doi: 10.3389/fendo.2022.873699 (PMC9329830; doi:10.3389/fendo.2022.873699)
Supplement: Supplementary file 1 [file Table_1.pdf]

Supplementary Table 1. Main charateristics of the organokines

	year discovered as oragnokine	Organokine type	expressed cells	expression in obesity	function in inflammation	mechasnism for regulation of inflammation
Leptin	1994	adipokine	primarily expressed in adipocytes	incrased	pro-inflammatory	enhance LPS-induced inflammation via activating NF-KB; JAK2/STAT3/CCLs; enhances phagocytosis
Resistin	2001	adipokine	in mouse adipocyts<br>in human PBMC and macrophages	increased	pro-inflammatory	upregulate proinflammtory cytokines in immune cells via NF-kB; resistin-TLR4 axis
Chemerin	2007	adipokine	adipocytes	incresaed	pro-inflammatory	chemokine for pDCs (recruit pDCs into viceral adipose tissue)
Progranulin	2012	adipokine	adipocytes, widely expressed	increased	pro-inflamamtory	induce macrophage chemotaxis, promote inflammation via SIRT1-NF-kB
RBP4	2007	adipokine	adipocytes, liver	increased	pro-inflammatory	prime macropahge TLR4/NLRP3
WISP1	2015	adipokine	widely expressed	increased	pro-inflammatory	promote macrophage M1 activation
FABP4	2006	adipokine	adipocytes	increased	pro-inflamamtory	reduce macrophage intraceeular unsaturated fatty acids, SIRT3 and UCP2, which activates inflammasome;<br>activate adipocyte p38/ NF-kB
PAI-1	1997	adipokine	broadly expressed	increased	pro-inflamamtory	promote M1 macropahge polarization
Follistatin-like 1	2010	adipokine	highly expression in SVF and preadipocytes	increased	pro-inflamamtory	avtivate NF-kB and NLRP3 inflammasome
MCP-1	2006	adipokine	adipocytes and SV fractions	increased	pro-inflamamtory	bind to CCR2 to promote macrophage infiltration into adipose tissue
SPARC	2001	adipokine	adipocytes	increased	pro-inflamamtory	increase expression of pro-inflammatory cytokines IL-6, MCP1 and TNF-α and<br>reduce anti-inflammatory cytokine IL-10 in adipocytes
SPARCL1	2016	adipokine	adipocytes and SV fractions	increased	pro-inflamamtory	induce liver inflammation, at least in part, through TLR4/NF-κB-dependent activation of MCP1
SAA	2006	adipokine	adipocytes	increased	pro-inflamamtory	endougenous ligand for TLR2 and TLR4
Adiponectin	1995	adipokine	mainly expressed in adipocytes	reduced	anti-inflammatory	promote M2 macropahge polarization via JMJD3-IRF4 axis
Omentin-1	2005	adipokine	visceral adipose tissue	reduced	anti-inflammatory	inhibit TXNIP/NLRP3 pathway (ref Zhou, 2020)<br>inhibit LPS-stimualted TLR4/ROS/NF-kB pathway (ref Wang 2020)
ZAG	2004	adipokine	adipocytes; liver	reduced	anti-inflamamtory	alleviate LPS-induced inflammation via β3-AR/PKA/CREB pathway in liver
SFRP5	2010	adipokine	adipocytes	reduced	anti-inflammatory	Sfrp5-JNK1 pathway
CTRP3	2008	adipokine	preadipocytes, adipocytes	reduced	anti-inflammatory	inhibit LPS/TLR4-mediated inflammation; inhibit NOD1 expression
LCN2	2007	adipokine	adipocytes, neutrophils,<br>macropahges, many cells	decreased in eWAT<br>increaed in serum	anti-inflammatory	promote PPARr in adipocytes and macrophages;<br>antagnize TNFa-induced inflammation and PPARr downregulation;<br>reduce LPS-induced M1 macrophage ploarization
VASPIN	2005	adipokine	visceral adipocytes	increased	anti-inflammatory	inhibit NF-kB in adipocytes
IL-10	2009	adipokine	adipose tisseu M2 macrophages	reduced	anti-inflammatory	suppress lipid-induced TNF-α production in macrophages and promote M2 macrophage polarization
IL-1RA	2003	adipokine	adipoes tissue	increaed	anti-inflammatory	bind to IL-1 receptor and antagonize the pro-inflammatory cytokine IL-1α and IL-1β
Fetuin A	2003	hepatokine	hepatocytes	increased	pro-inlfammatory	bind with FFA and TLR4 to activate NF-kB; promote macropahge M1 polarization via C-Jun and JNK-mediated IFNr
DPP4	2011	hepatokine /adipokine	hepatocytes, adipocytes, broad expression	increased	pro-inlfammatory	induce T cell activation and proliferation; activate NF-kB in macropahges and adipocytes
FGF21	2000	hepatokine	hepatocytes	increased	anti-inflamamtory	inhibit LPS-induced inflammation in adipocytes and macrophages via Nrf2/HO-1 and suppress NF-kB
GDF15	2018	hepatokine	hepatocytes (main), adipocytes	increased	anti-inflammatory	promote macrophage toward to a M2 polarization via upregulating oxidative metabolism
MANF	2021	hepatokine	hepatocytes, endocrine cells	increased	anti-inflammatory	attenuate inflammation is via binding to p65 via its C-terminal SAP-like domain and suppress NF-κB activation
Irisin	2012	myokine /adipokine	exercised skeletal muscle, adipocytes	increased	anti-inflamamtory	suppress inflamamtory response via TLR4/MyD88-mediated NF-κB and NLRP3 inflammsom
IL-6	2000	myokine	exercised skeletal muscle	increased	anti-inflamamtion	IL-6/STAT3 singling promotes M2 macrophage polarization and proliferation via inducing IL-4R expression
DEL-1	2020	myokine	exercised skeletal muslce	reduced	anti-inflamamtory	reduce NF-κB activation and pro-inflammatory cytokines secretion via activating AMPK/HO-1 signaling in adipocytes
